# Antifungal Activity and Biosynthetic Potential of New *Streptomyces* sp. MW-W600-10 Strain Isolated from Coal Mine Water

**DOI:** 10.3390/ijms22147441

**Published:** 2021-07-12

**Authors:** Piotr Siupka, Frederik Teilfeldt Hansen, Aleksandra Schier, Simone Rocco, Trine Sørensen, Zofia Piotrowska-Seget

**Affiliations:** 1 Faculty of Natural Sciences, Institute of Biology, Biotechnology and Environmental Protection, University of Silesia in Katowice, 40032 Katowice, Poland; aleksandra.kaszyca@us.edu.pl (A.S.); simone.rocco@edu.unito.it (S.R.); zofia.piotrowska-seget@us.edu.pl (Z.P.-S.); 2 Faculty of Engineering and Science, Department of Chemistry and Biosciences, University of Aalborg, 9220 Aalborg, Denmark; jft@bio.aau.dk (F.T.H.); trso@bio.aau.dk (T.S.)

**Keywords:** antifungal activity, biosynthetic gene clusters, genome mining, coal-related environments, *Streptomyces*, quantitative PCR, gene expression

## Abstract

Crop infections by fungi lead to severe losses in food production and pose risks for human health. The increasing resistance of pathogens to fungicides has led to the higher usage of these chemicals, which burdens the environment and highlights the need to find novel natural biocontrol agents. Members of the genus *Streptomyces* are known to produce a plethora of bioactive compounds. Recently, researchers have turned to extreme and previously unexplored niches in the search for new strains with antimicrobial activities. One such niche are underground coal mine environments. We isolated the new *Streptomyces* sp. MW-W600-10 strain from coal mine water samples collected at 665 m below ground level. We examined the antifungal activity of the strain against plant pathogens *Fusarium culmorum* DSM62188 and *Nigrospora oryzae* roseF7. Furthermore, we analyzed the strain’s biosynthetic potential with the antiSMASH tool. The strain showed inhibitory activity against both fungi strains. Genome mining revealed that it has 39 BGCs, among which 13 did not show similarity to those in databases. Additionally, we examined the activity of the *Streptomyces* sp. S-2 strain isolated from black soot against *F. culmorum* DSM62188. These results show that coal-related strains could be a source of novel bioactive compounds. Future studies will elucidate their full biotechnological potential.

## 1. Introduction

Fungal pathogens are a burden for agriculture worldwide. Many fungal strains that infect important crops cause severe losses in food production [1]. Members of the *Fusarium* genus are particularly known for their pathogenicity against crops [2,3]. *F. graminearum* and *F. culmorum* are, among others, pathogens of major cereals, including wheat and maize, and can cause fusarium head blight, fusarium foot rot, and fusarium crown rot [4,5]. *Fusarium* strains are also responsible for banana wilt-disease, one of the most devastating plant diseases known [6,7]. The fungal infection of plants poses an additional danger to humans and livestock as they produce a variety of toxins that contaminate agriculture food products [8,9,10,11,12]. Extensive use of existing fungicides can be harmful to the environment as their accumulation might have adverse effects on other species of fungi, plants, and animals. The efficient application of fungicides also depends on weather conditions [13]. Additionally, the resistance to existing compounds by pathogenic fungal strains is on the rise. All these have led researchers to look for natural control agents or substances of natural origin that could be used to fight fungal infections.

The *Actinobacteria*, especially members of the genus *Streptomyces*, are known for their ability to produce a plethora of secondary metabolites with biological activities, including antifungal compounds [14,15,16]. These Gram-positive bacteria generally have a high GC content, large genomes, and complex morphology, and they are commonly found in soil and water environments. It is estimated that they may produce over 100,000 compounds with antibiotic, antitumor, or antifungal activities, among others [17,18]. Most antibiotics developed in the 21st century are based on the *Streptomyces* secondary metabolites [19,20]. Many *Streptomyces* have antifungal properties [21,22]. A number of strains have biocontrol potential for *Magnaporthe oryzae* (*Pyricularia oryzae*), a fungus causing rice blast [23]. Another strain, *Streptomyces* sp. SCA3-4, isolated from rhizosphere soil of a cactus—erect prickly pear (*Opuntia stricta*)—has been shown to have a broad-spectrum antifungal activity [24].

The advancement and increased accessibility of molecular tools, especially whole-genome sequencing, allows for better identification of the new strains and evaluation of their biosynthetic potential [25,26]. It is possible thanks to the development of next-generation sequencing methods in the 21st century. The Illumina dye sequencing provides a large number of short, high-precision sequences, while Nanopore sequencing generates long reads, albeit with a higher error rate [27,28]. The combination of both methods for microbial strains analysis leads to high-quality genomes [29,30,31]. Phylogeny based on the whole-genome sequencing and comparison of multiple loci provides a more precise identification compared to standard 16S rRNA gene analysis, especially for microorganism’s taxa where a large number of strains have been studied [32,33]. Moreover, the sequenced genome enables a number of other analyses, including the evaluation of biosynthetic potential [34,35,36]. Genome mining has shown that *Streptomyces* encompass between 8 and 83 biosynthetic gene clusters (BGCs), indicating a large untapped biosynthetic potential [26]. At the same time, many of the clusters are dormant or not expressed under laboratory conditions [37,38,39,40]. Therefore, in the last decade, almost no new classes of antibiotics have been discovered [41], and most of the attempts have resulted in the isolation of already-known strains or compounds [26,42]. For that reason, the focus of research has switched to extreme or nonconventional niches. *Streptomyces* strains with antimicrobial properties were isolated among others from artic soil, coal mine soil, black soot after hard coal combustion, caves, insects, and marine sponges [34,43,44,45,46,47]. They have been found to be sources of new compounds [46]. It has been shown that strains of the same species of *Streptomyces* have a variable number of BGCs [26,35,48,49,50]. Moreover, strains from extreme environments tend to have a higher number of BGCs than their terrestrial counterparts [45].

In the current study, we aimed to examine antifungal properties and the biosynthetic potential of a new *Streptomyces* strain isolated from coal-related environments. We also attempted to obtain clues about metabolites involved in the antifungal activity and the influence of fungi on the expression of BGCs in *Streptomyces* from coal-related environments. We performed core proteome-based phylogenetical analysis of the *Streptomyces* sp. MW-W600-10 strain isolated from collective coal mine water, from depths 665–850 m below ground level (bgl) sampled at the coal mine pump station at 665 m bgl in Upper Silesia, Poland. The core proteome was predicted based on a high-quality genome obtained through combined Nanopore and Illumina sequencing. We evaluated antifungal properties of the strain by testing the influence the bacteria have on the growth of two filamentous fungi, both plant pathogens—*F. culmorum* DSM62188 and environmental isolate *N. oryzae* roseF7 [43]. Additionally, we tested the activity of a previously described strain isolated from coal-related environments (CREs), *Streptomyces* sp. S-2 [43], on *F. culmorum* DSM62188. The genome of MW-W600-10 was sequenced and mined for BGCs to elucidate its strain biosynthetic potential. We compared it to other *Streptomyces* strains and revised the BGCs content of *Streptomyces* sp. S-2. Finally, we attempted to obtain insight into the strains’ response to fungus strains by studying the change in expression levels of selected biosynthetic gene clusters. This study points to CREs as a source of new potential biocontrol agents.

## 2. Results

### 2.1. Isolation of Streptomyces sp. MW-W600-10 Strain 

The MW-W600-10 strain was isolated on a potato dextrose agar (PDA) plate and showed an *Actinobacteria*-like morphology (Figure S1). On PDA, it grew as compact, creamy, brown-bottom colonies with radiant exploratory mycelium observed within the medium and light brown staining of the culture medium. On the Mueller–Hinton agar plate (MHA), it grew as compact, rough, and transparent, which, over time, turned into creamy white colonies with observed exploratory mycelium growing into the medium. The strain was selected for further studies.

### 2.2. Genome Sequencing and Phylogeny of Streptomyces sp. MW-W600-10

**Figure 1:**
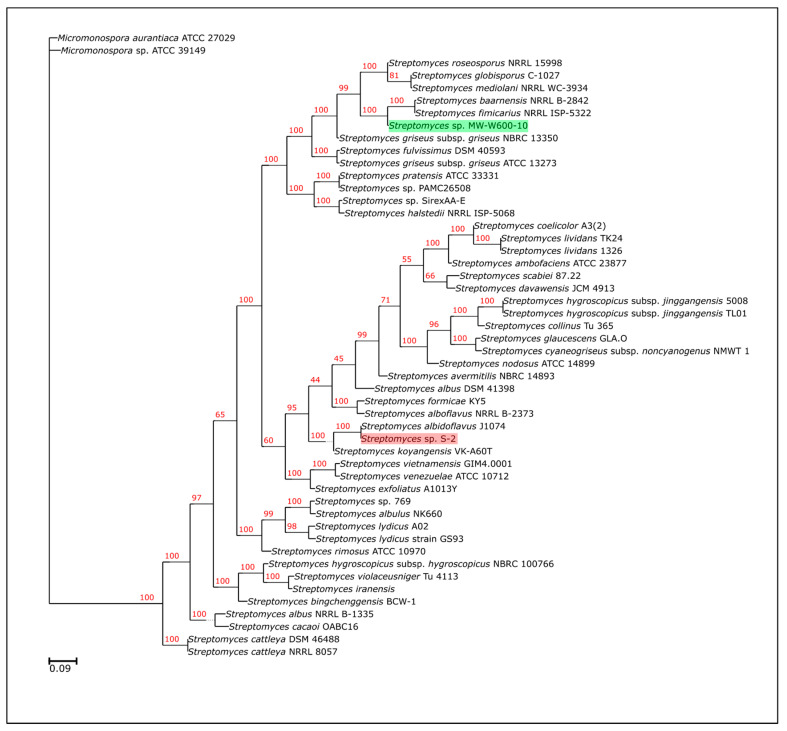
Maximum-likelihood phylogenetic tree of the *Streptomyces* sp. MW-W600-10 strain. The tree shows the phylogenetic relation between MW-W600-10 and other Streptomyces strains. The tree was calculated using IQ-TREE software based on the alignment of core proteomes of 50 *Actinobacteria* strains, and *Micromonospora* strains were used as an outgroup. Numbers at the nodes in red represent bootstrap values (% of 1000 repeats). The position of MW-W600-10 has been highlighted in green; the position of another coal-related strain, *Streptomyces* sp. S-2, has been highlighted in red for better visibility.

The genome sequencing showed that the MW-W600-10 strain (GenBank accession number: JAGTPS000000000) has 8.37 Mbp, 71.69% G + C content genome, and 7362 identified CDSs. Statistics for the assembled genome of MW-W600-10 and the revised assembly of the S-2 strain (GenBank accession number: WMKI00000000, version WMKI02000000) are shown in Table 1. Both genomes have been used for phylogeny analysis, which included 49 other *Actinobacteria* strains, 47 *Streptomyces*, as well as *Micromonospora aurantiaca* ATCC 27,029, and *Micromonospora* sp. ATCC39149 as an outgroup. The maximum-likelihood phylogenetic tree generated based on the strain’s core proteomes showed that the closest relatives of the MW-W600-10 strain are *S. fimicarius* NRRL ISP-5322 and *S. baarnensis* NRRL B-2842 (Figure 1). Importantly, the phylogeny of the *S. fimicarius* NRRL ISP-5322 strain was revised recently, and it was suggested that the strain should be classified as *S. setonii* [51]. Here, we kept the taxonomical name of the NRRL ISP-5233 strain displayed in the NCBI database. Based on that, we suggest that the MW-W600-10 strain is a new isolate of *Streptomyces* most closely related to *S. fimicarius*/*S. setonii* and *S. baarnensis*.

### 2.3. Antifungal Activities of Streptomyces from CRE

**Figure 2:**
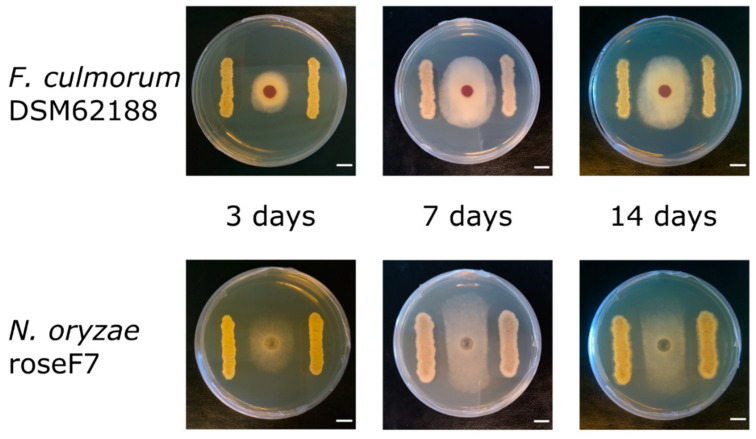
Inhibition of fungal growth by *Streptomyces* sp. MW-W600-10. Representative pictures of the MW-W600-10 strain in co-culture with both examined fungi on MHA plates. Pictures from 3 day *Streptomyces* pre-culture with MW-W600-10 inoculated at 25 mm from the center of the plates. An 8 mm agar puck with fungal mycelium was placed at the plate center. Pictures were taken at the 3rd, 7th, and 14th day of co-culture. The scale bar represents a distance of 10 mm.

**Figure 3:**
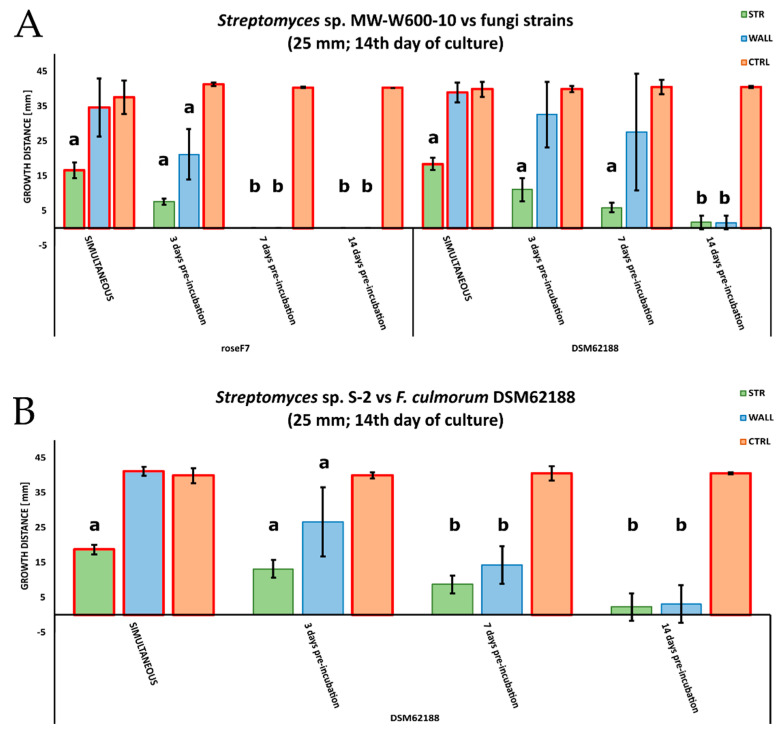
Inhibition of fungal growth by coal-related *Streptomyces* strains. (**A**) Effect of co-culture with MW-W600-10 strain on the growth of *Nigrospora oryzae* roseF7 and *Fusarium culmorum* DSM62188; (**B**) effect of co-culture with S-2 strain on the growth of *F. culmorum* DSM62188. The charts compare the growth of fungal mycelium toward *Streptomyces* streak colonies (STR), the plate wall (WALL), and fungal growth on control plates (CTRL) for different co-cultures where *Streptomyces* were inoculated at 25 mm from the center of the plate. Results are shown for the 14th day of co-culture. For each experimental setup, 5 biological repeats were performed for co-cultures and 3 repeats for controls. The red border of the bars indicates the fungus reaching the plate wall or bacterial streaks by the day of measurement. Error bars represent the standard deviation of mycelial growth in independent biological experiments. Statistically significant differences between measurements analyzed for each experimental setup separately are marked by the (a) difference between all measurements or by the (b) difference between measurement and CTRL only (one-way ANOVA, followed by Tuckey’s post hoc test; *p* ≤ 0.05).

**Figure 4:**
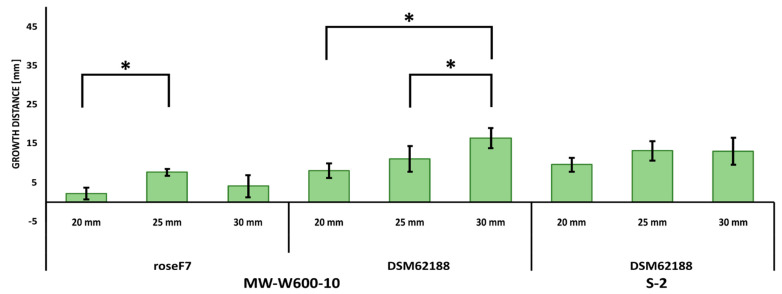
Influence of the distance of the inoculation of *Streptomyces* on the inhibition of fungi growth. The results present the growth toward the *Streptomyces* streak at the 14th day of co-culture with bacteria pre-incubated for 3 days. For each experimental setup, 5 biological repeats were performed. Error bars represent the standard deviation of mycelial growth in independent biological repeats. Statistically significant differences between measurements analyzed for each experimental setup separately are marked by “*” (one-way ANOVA, followed by Tuckey’s post hoc test; *p* ≤ 0.05).

**Figure 5:**
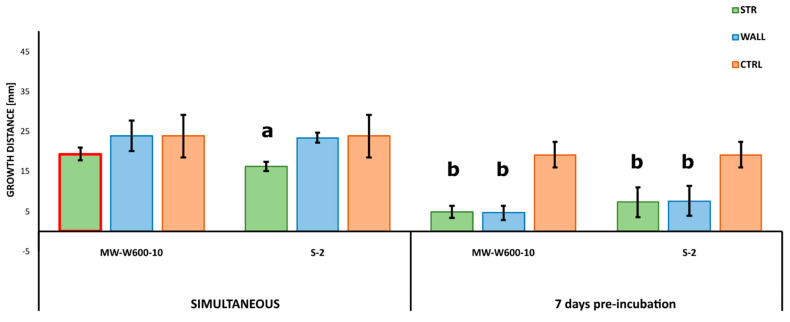
Different effects of *Streptomyces* sp. MW-W600-10 and *Streptomyces* sp. S-2 on the growth of *F. culmorum* DSM62188 in co-cultures—simultaneous and with bacteria pre-incubated for 7 days. The charts compare the growth of fungal mycelium toward *Streptomyces* streak colonies (STR), the plate wall (WALL), and fungal growth on control plates (CTRL) for co-cultures with *Streptomyces* inoculated at a 25 mm distance from the center of the plate. Results are shown for the 3rd day of co-culture. For each experimental setup, 5 biological repeats were performed for co-cultures and 3 repeats for controls. The red border of the bars indicates the fungus reaching the plate wall or bacterial streaks by the day of measurement. Error bars represent the standard deviation of mycelial growth in independent biological experiments. Statistically significant differences between measurements analyzed for each experimental setup separately are marked by the (a) difference between all measurements or by the (b) difference between the measurement and CTRL only (one-way ANOVA, followed by Tukey’s post hoc test; *p* ≤ 0.05).

The antifungal activity was tested by measuring the inhibition of fungal growth by *Streptomyces* using a plate assay. The activity of the MW-W600-10 strain was tested against plant pathogens *Fusarium culmorum* DSM62188 and *Nigrospora oryzae* roseF7. Additionally, we tested the activity of *Streptomyces* sp. S-2 isolated from black soot after hard cold combustion [43], against *F. culmorum* DSM62188. *Streptomyces* strains were co-cultured with fungi on PDA plates in a simultaneous co-culture or with pre-incubation for 3, 7, or 14 days. Additionally, for each type of co-culture, *Streptomyces* strains were inoculated at distances of 20, 25, or 30 mm from the fungi inoculation spot. The MW-W600-10 strain showed activity against both fungi, albeit with different levels of inhibition. Representative pictures of the inhibition of fungal growth by the MW-W600-10 strain are shown in Figure 2. The comprehensive fungal growth inhibition by *Streptomyces* strains is shown in Figure S2. The MW-W600-10 strain exhibited a more robust activity against *N. oryzae* roseF7 than *F. culmorum* DSM62188 (Figure 3A). The activity was strongest in pre-incubated co-cultures with the 7 day and 14 day pre-culture of MW-W600-10 resulting in the almost complete inhibition of *N. oryzae* roseF7 and *F. culmorum* DSM62188 growth, respectively. Under all conditions, the growth of fungi toward *Streptomyces* sp. MW-W600-10 was inhibited compared to the growth of fungi on control plates. In particular cases, the growth toward the strain was more inhibited than the growth toward the plate wall (Figure 3). However, for the simultaneous co-culture, *F. culmorum* DSM62188 was already able to reach bacteria colonies at day 3 of co-culture except for the streaks inoculated at the distance of 30 mm from the plate center. This most likely influenced the measurements (Figure S2). In other experimental conditions, the fungi did not reach the bacterial colonies. Under most conditions, the distance of the inoculation did not significantly influence inhibition, although a trend of less inhibition with greater distance was observed (Figure 4). That is not too surprising considering that the active substances have to diffuse through the medium. The *Streptomyces* sp. S-2 was also able to inhibit the growth of *F. culmorum* DSM62188 with a 14 day pre-culture resulting in the almost complete inhibition of fungal growth (Figure 3B). In simultaneous co-cultures, the fungi did not reach the bacteria streaks in any of the setups, suggesting stronger inhibitory properties of S-2 than MW-W600-10 at the beginning of the culture. However, in pre-cultures, fungal growth toward *Streptomyces* was less inhibited than in MW-W600-10 pre-cultures. That was pronounced most for the 7 day preincubation of *Streptomyces* strains (Figure 5). At the same time, there was no stronger inhibition of fungal growth toward the plate wall by S-2 in co-cultures at a distance of 30 mm compared with co-cultures at 20 and 25 mm in the 3 day preincubation setup. Stronger inhibition was observed in the 7 day preincubation setups between distances of 20 and 30 mm. The results suggest that antifungal compounds produced by both strains have different expression profiles.

### 2.4. Putative Biosynthetic Gene Clusters Prediction in the Streptomyces sp. MW-W600-10 Strain 

**Figure 6:**
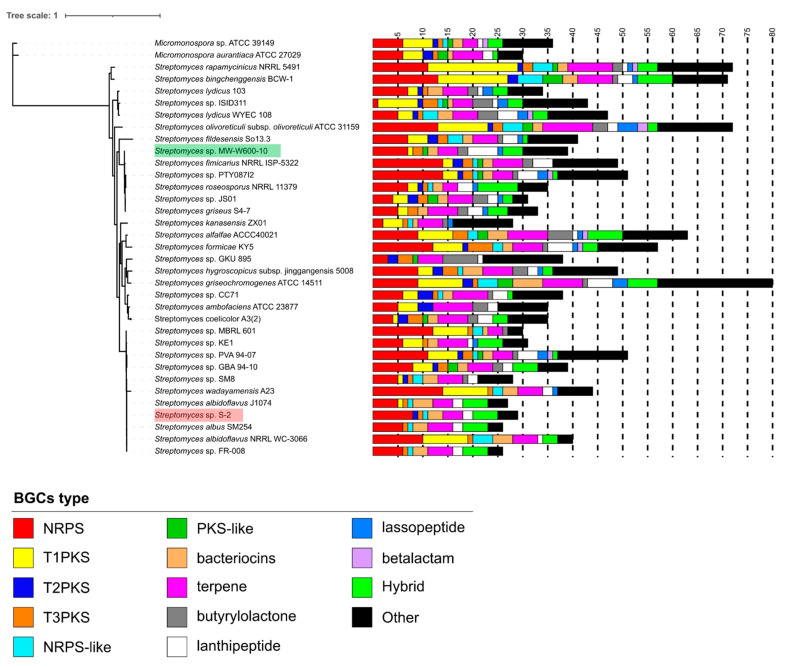
Comparison of number of BGCs in genomes of closely related *Streptomyces* strains and strains reported to have a high number of clusters. The BGCs were predicted using the antiSMASH tool, the results were manually investigated, “neighboring” clusters were not counted, and “interleaved” clusters were counted as separate clusters. Clusters not belonging to the shown groups were pooled in the “other” group. The maximum likelihood tree was generated based on the alignment of 18 single-copy genes found in each strain, where positions of coal-related strains have been highlighted for better visibility—MW-W600-10 in green and S-2 in red.

The genome of the MW-W600-10 strain was used for the prediction of secondary metabolites biosynthetic gene clusters using antiSMASH. Additionally, the revised genome of the S-2 strain was re-analyzed with the same version of the tool. Results were manually investigated to provide precise information on predicted clusters. A full list of the antiSMASH results can be found in Table S1. The analysis predicted 39 clusters encoded in the MW-W600-10 genome, among which 13 of them do not show similarities to known clusters present in the databases, and 12 show similarities below 20% (Table S1). It is most noticeable that seven clusters belong to nonribosomal peptide synthetases (NRPS), four to polyketide (PKS) or polyketide-like (PKS-like) synthetases, six to lanthipeptides, and two to bacteriocins, and there are also one heterocyst glycolipid synthase-like polyketide synthase (hgIE-KS) and two butyrolactone clusters. There are also four hybrid clusters, out of which three are NRPS-T1PKS and one is a LAP-thiopeptide hybrid (Figure 6 and Table 2). The number of clusters shows various levels of similarity to known antimicrobial or cytotoxic compounds (Table S1). Among others, typical clusters for *Streptomyces* secondary metabolites are present—geosmin and desferrioxamine B.

For the S-2-revised genome, the analysis detected 28 clusters as opposite to the previous yield of 55 clusters. The most common types of clusters were NRPSs—eight clusters, and there are also five hybrid clusters, among which four are NRPS-T1PKS hybrids. Eight clusters do not show similarities to those present in the antiSMASH database.

The number of predicted clusters in *Streptomyces* from CRE was compared to those found in other *Streptomyces* strains (Figure 6 and Table 2). The MW-W600-10 strain has an average number of BGCs for the *Streptomyces* genus (39 vs. 39.64 ± 11.40 clusters), while the S-2 strain has a lower number. On the other hand, the S-2 strain is enriched in NRPS clusters, where they comprise 27.59% of the strain’s BGCs with an average of 18.49% for other strains used in the analysis. For MW-W600-10, 17.95% of its clusters belong to NRPS, yet this group is still the most represented in the strain’s genome. At the same time, the closest relative to MW-W600-10, *S. fimicarius* NRRL ISP-5322, has an additional 10 clusters (39 vs. 49 for MW-W600-10 and NRRL ISP-5322, respectively). It also has more NRPS (7 vs. 14), NRPS-like (0 vs. 1), T1PKS (1 vs. 2) and T2PKS (0 vs. 2), hgIE-KS (1 vs. 2), terpenes (4 vs. 6), and clusters grouped as other (2 vs. 7). On the other hand, MW-W600-10 strains have more lanthipeptides (6 vs. 4), melanin (1 vs. 0), lasso peptide (1 vs. 0), and hybrid clusters (4 vs. 0). The number of clusters with no similarities to any in the database is similar between two strains, 13 vs. 14 for MW-W600-10 and NRRL ISP-5322, respectively. However, they are distributed differently across different types of BGCs. A direct comparison of predicted BGCs in CRE strains and their closest counterparts is shown in Table 2. These results show that strains from coal-related environments can be a potential source on novel antimicrobial compounds.

### 2.5. Expression of BGCs in Streptomyces from CRE

**Figure 7:**
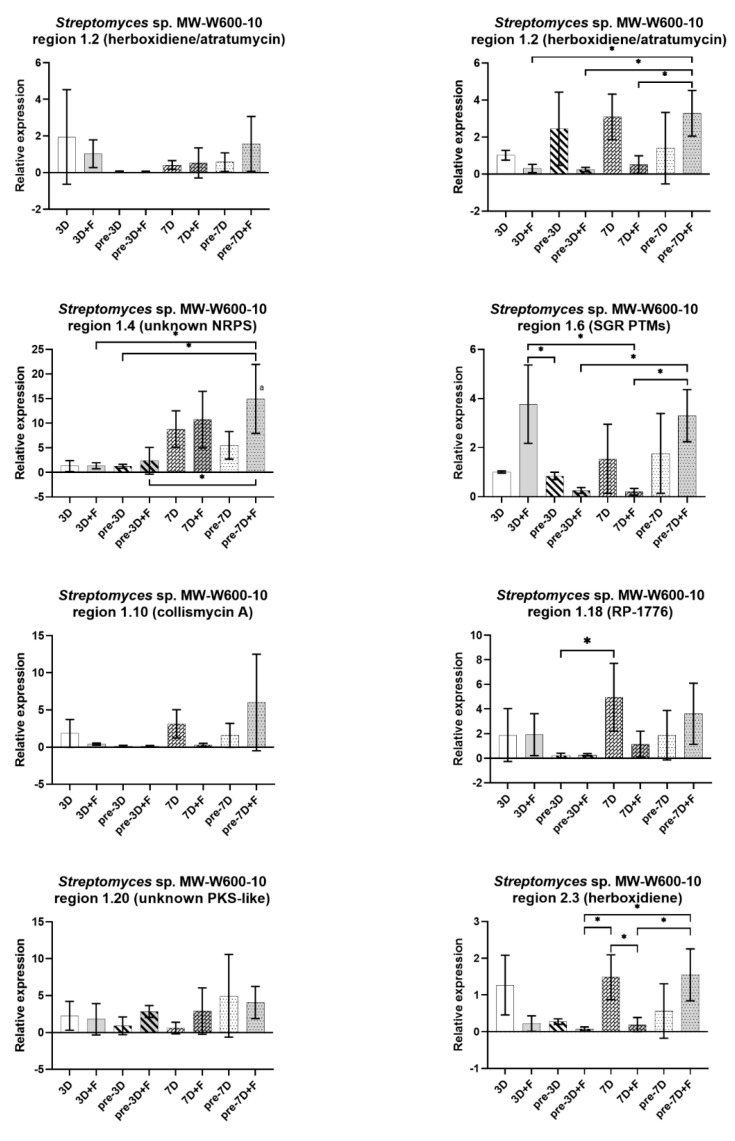
Relative expression of selected BGCs of *Streptomyces* sp. MW-W600-10 grown as a single culture (3D, pre-3D, 7D, and pre-7D) or in co-culture with *F. culmorum* DSM62188 (3D + F, pre-3D + F, 7D + F, and pre-7D + F). The strain was cultured in simultaneous (3D and 7D) cultures or with the pre-cultured *Streptomyces* strain for 3 (pre-3D) or 7 (pre-7D) days. The qPCR was performed on mRNA samples purified from cultures at the 3rd (3D; 3D + F), 6th (pre-3D; pre-3D + F), 7th (7D; 7D + F), and 14th (pre-7D; pre-7D + F) day of incubation. For each sample, two culture plates were used for mRNA purification, and each experimental setup comprised at least 3 biological repeats. The charts show mean values and standard deviations of the expression ratio relative to the control (average of 3D samples) after the removal of outliers. The scale has been adjusted for each cluster separately for better visibility. Statistically significant differences in relative expression levels in different experimental setups are marked by ‘a’ for expression levels significantly different from the expression in the 3D setup; *—significant difference between indicated setups (one-way ANOVA followed by Tukey’s post hoc test, *p* ≤ 0.05).

**Figure 8:**
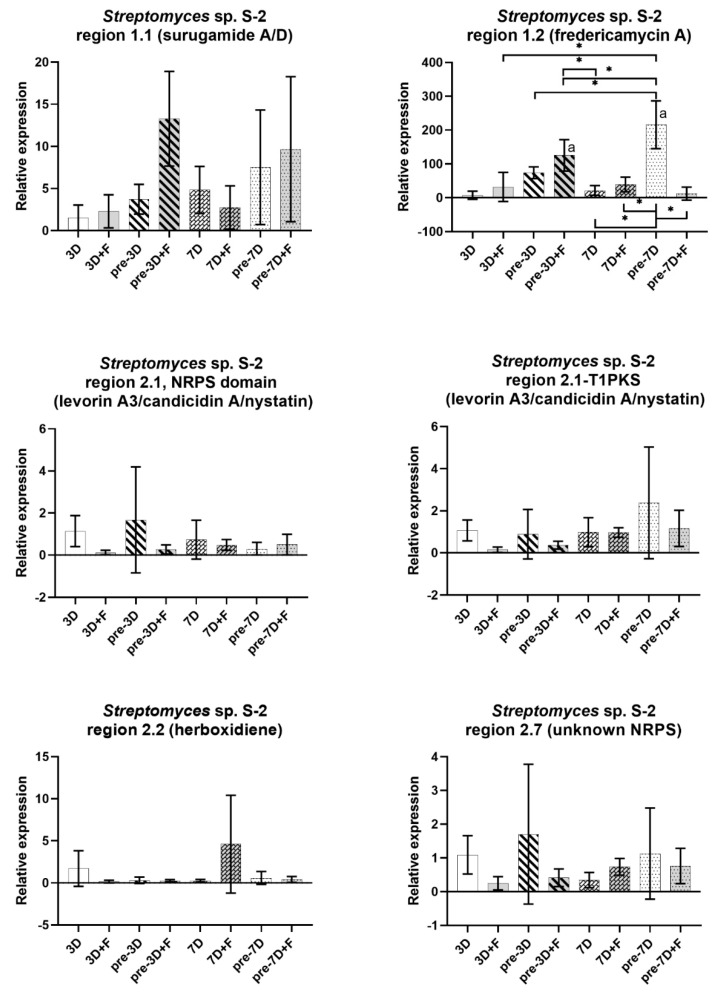
Relative expression of selected BGCs of *Streptomyces* sp. S-2 grown as a single culture (3D, pre-3D, 7D, and pre-7D) or in co-culture with *F. culmorum* DSM62188 (3D + F, pre-3D + F, 7D + F, and pre-7D + F). The strain was cultured in simultaneous (3D and 7D) cultures or with the pre-cultured *Streptomyces* strain for 3 (pre-3D) or 7 (pre-7D) days. The qPCR was performed on mRNA samples purified from cultures at the 3rd (3D; 3D + F), 6th (pre-3D; pre-3D + F), 7th (7D; 7D + F), and 14th (pre-7D; pre-7D + F) day of incubation. For each sample, two culture plates were used for mRNA purification, and each experimental setup comprised at least 3 biological repeats. The charts show mean values of the expression ratio relative to the control (average of 3D samples) after the removal of outliers. The scale has been adjusted for each cluster separately for better visibility. Statistically significant differences in relative expression levels in different experimental setups are marked by ‘a’ for expression levels significantly different from the expression in the 3D setup; *—significant difference between indicated setups (one-way ANOVA followed by Tukey’s post hoc test, *p* ≤ 0.05).

The expression of selected BGCs for each of the strains was tested by the employment of real-time quantitative polymerase chain reaction (RT-qPCR) in the *Streptomyces* monoculture (3D, pre-3D, 7D, and pre-7D) or in co-culture with *F. culmorum* DSM62188. Simultaneous (3D + F and 7D + F) and co-cultures with pre-cultured *Streptomyces* (pre-3D + F and pre-7D + F) were used. The RNA was isolated at the 3rd, 6th, 7th, and 14th day of growth. The expression of the clusters was compared with the average expression from the monoculture after 3 days of incubation. The expression of RNA polymerase principal sigma factor gene, *hrdB*, was used as a reference. Clusters belonging to NRPS or PKS that showed similarities to potentially antifungal compounds, as well as no similarity to known clusters, were chosen for analysis. A list of clusters for each strain and the level of similarity, together with primers used for RT-qPCR, are shown in Table S4. For each strain, the expression profiles of the cluster varied between cultures. There were large variations between biological repeats, because of that most results did not show statistical significance. Nevertheless, trends can be observed. Charts presenting results for MW-W600-10 and S-2 strains are shown in Figure 7 and Figure 8, respectively. For MW-W600-10, during the course of the experiment, an upregulation of an unknown NRPS cluster in region 1.4 was observed. The presence of fungus in the cultures did not change the expression significantly, although a slight but not significant increase was observed for the simultaneous co-culture after 7 days and the co-culture with the pre-cultured strain for 7 days (14th day of *Streptomyces* culture and 7th of co-culture). The fungal presence also caused an upregulation of the cluster in region 1.6 in the simultaneous co-culture at day 3 (3D + F) and a potential upregulation in the co-culture with the pre-cultured strain (pre-7D + F). On the other hand, in the rest of the co-cultures, region 1.6 seems to be downregulated. Fungi presence seems to cause the downregulation of this and others of the analyzed clusters in regions 1.10 (collismycin A), 1.20 (unknown PKS), and 2.3 (herboxidiene). Peculiarly, for the cluster in region 1.2, two core biosynthetic genes from the same cluster were targeted and showed different expression profiles. One of the genes seemed to be downregulated during the course of the culture (in both mono- and co-culture), while the other gene exhibited a slight upregulation during the culture. However, it was downregulated when the fungus was present (except pre-7D + F setup; however, the variations were high).

For the S-2 strain, an upregulation of the cluster was observed in region 1.1, showing similarity to the surugamide A/D cluster. That was most prominent in the co-culture with fungus and pre-culture S-2 for 3 days (3rd day of co-culture, 6th day of *Streptomyces* culture; pre-3D + F). An even more prominent upregulation was observed for the cluster in region 1.2 showing similarity to the fredericamycin A cluster. Here, the expression appears stimulated by fungus already in the simultaneous co-culture, with a continually increased expression at the 6th day of *Streptomyces* culture (pre-3D), which seems to be further stimulated by fungus (pre-3D + F). The highest expression for that cluster was observed at the 14th day of culture (pre-7D) and, surprisingly, in the corresponding co-culture, the presence of fungus seems to hamper the expression (pre-7D + F). Nevertheless, it was still 12 times higher than in the reference setup, but with very high variations between repeats (data not shown). For the rest of the clusters analyzed, there was no difference in the expression, or the expression was reduced either in the course of the culture or in the presence of fungus. This was most noticeable for the cluster in region 2.2 (similarity to herboxidiene). It was severely downregulated in all experimental setups, except the simultaneous co-culture with fungus after 7 days (7D + F), but the results for this point were highly variable. The cluster in region 2.1 predicted to encode the NRPS-T1PKS hybrid was targeted for the expression of core biosynthetic genes either for the NRPS or T1PKS region. Both genes seem to be downregulated when fungus is present. When the strain was grown as a monoculture, they showed different patterns of regulation. The NRPS gene showed a slight upregulation in bacteria pre-cultured for 3 days, while the T1PKS gene in bacteria pre-cultured for 7 days. However, the variance was high for both genes and the results were not statistically significant.

## 3. Discussion

Coal mines and coal-related environments have been suggested as a potential source of new compounds and strains with antimicrobial properties [52]. In fact, *Streptomyces* strains showing antimicrobial activities were isolated before from coal-related environments [43]. Here, we describe a new *Streptomyces* sp. MW-W600-10 strain that was isolated from a coal mine shaft’s collective water (from depth 665–850 m bgl) sampled at a depth of 665 m bgl in a coal mine in Upper Silesia, Poland. The strain genome was sequenced, showing that it contains 8.43 Mbp, 71.70% G + C content, and 7738 CDS, typical for the *Streptomyces* genus that, on average, has 8.6 Mbp genomes with >7000 CDS [17,44]. Phylogenetic analysis based on the core proteome showed that the strain is closely related to *S. fimicarius* NRRL ISP-5322. It should be noted that *S. fimicarius* was suggested to be reclassified as a synonym of *S. setonii* [51]. That is peculiar as *S. setonii* 75Vi2 has been shown before by Strandberg and Lewis [53] to be able to solubilize coal. Bearing that in mind, the occurrence of the MW-W600-10 strain in coal mine environments is not surprising. It would be interesting to see if the new strain is able to bioprocesses coal or coal-related contaminations. This is something worth investigating in the future research.

We investigated the antifungal properties of the MW-W600-10 strain against two filamentous fungi, both plant pathogens—*F. culmorum* DSM62188 and *N. oryzae* roseF7. The strain showed activity against both fungi, albeit with different levels of inhibition. The inhibition effect was stronger with the pre-incubation period of *Streptomyces*. The stronger activity was against *N. oryzae* roseF7, with an almost complete abolishment of fungal growth in the 7-day pre-incubation co-culture as opposed to *F. culmorum* DSM62188, where complete inhibition was observed for the 14-day pre-cultured assays. There was a trend of inhibition change with the distance of *Streptomyces* inoculation from the fungal inoculation. The longer the distance, the smaller the inhibition of the fungal growth, which is not surprising, as the produced compounds need to diffuse through the media; however, those differences were, in most cases, not statistically significant. Previously, another CRE strain, isolated from the black soot after hard coal combustion, *Streptomyces* sp. S-2, has been shown to have strong antifungal activity against a number of filamentous fungi, including *N. oryzae* roseF7 [43]. Here, we tested its activity against *F. culmorum* DSM62188. The results were similar to those for MW-W600-10. However, in simultaneous and 3-day pre-incubation setups, the distance had a significant influence on the inhibition. That points to the early production of fungicides by the strains. Interestingly, when two strains were compared, the S-2 had a slightly higher activity in the simultaneous co-culture, while MW-W600-10 showed a higher inhibition of fungal growth in pre-culture assays. The S-2 strain also showed a slightly higher inhibition of the growth of fungal mycelium toward the plate walls than MW-W600-10. One possible explanation for these might be the different expression profiles of the SMs produced by the two strains. S-2 might produce antifungal compounds early on during culture, while MW-W600-10 might activate the production in later phases of growth. Other studies have shown antifungal activity of *Streptomyces* isolated from previously omitted niches and extreme environments. Besides the S-2 strain mentioned above, Maciejewska et al. [54] examined the activity of *Streptomyces* isolated from cave moonmilk deposits. The strains had antimicrobial activity against a number of bacterial and fungal genera [54]. Insect symbionts belonging to *Streptomyces* were shown to possess a wide range of antimicrobial activities, including activity against fungi *Candida* sp., *Aspergillus flavus*, *Cryptococcus neoformans*, *Rhizopus oryzae*, *Saccharomyces cerevisae*, *Trichoderma* sp., and *Metarhizium* sp. [46]. Coal-related environments could also be a source of strains with antifungal activity. Kim et al. [55] isolated *Streptomyces fodineus* from coal mine soil, showing activity against a number of pathogenic fungi. Increasing attention has been given to the use of *Streptomyces* as biocontrol agents to fight crop diseases [7,56,57,58,59]. *S. lydicus* WYEC 108 is the active compound in fungicide used in the USA [60]. A number of studies have focused on the potential use of *Streptomyces* to fight banana wilt-disease [7,61]. It would be interesting to see if both strains from CRE could be used as biocontrol agents in agriculture. This is a topic that should be investigated further in the future.

Genome mining has shown that MW-W600-10 has 39 putative BGCs, among which 13 show no similarity to those found in databases. A large portion of clusters have a similarity to these below 20% in databases. This indicates that the SM they encode could display a novel activity [45]. The BGCs number is an average for the *Streptomyces* genus (approx. 39 clusters per strain) [26], but it is greater than the number of BGCs for soil strains, which usually contain 20–30 clusters [44,62]. The number of putative BGCs in the MW-W600-10 genome is higher than in other strains isolated from extreme environments [44,45], including *Streptomyces* sp. S-2 isolated from black soot [43]. The number of BGCs predicted in the MW-W600-10 genome is, however, smaller than in its closest neighbor, *S. fimicarius* NRRL ISP-5322. It is important to note that the sequencing method and resulting lower number of contigs could have an influence on the number of predicted BGCs. In the current study, we used long-read Nanopore sequencing combined with high-precision Illumina sequencing. This approach allowed us to obtain high-quality genomes in which the BGCs do not span multiple contigs, making accurate prediction by antiSMASH more reliable. For the same reason, the number of putative clusters in the S-2 strain was reduced almost by 50% compared to the previous report where only Illumina reads were used [43]. Still, the most represented group among the predicted cluster was NRPSs, which has been shown to be typical for the genus [26]. Despite the MW-W600-10 and previously described S-2 strains not being new species, their potential to be used as biocontrol agents or producers of novel bioactive compounds should not be disregarded. Recently, a number of studies has shown that biosynthetic potential is strain-dependent rather that species-dependent [26,35]. Analysis of the BGCs distribution among 18 strains of *S. lunelactis* has shown that some of the clusters are present only in a few or a singular strain, while others are widely distributed [48]. Similarly, two works by Komaki et al. [49,50] showed the diversity of NRPS and PKS in taxonomically closely related *Streptomyces*. The ecology of the microorganism, niche, or geographical location from where it was isolated can also have an impact on the biosynthetic potential [46,63,64]. 

Regarding the clusters encoding compounds with antifungal activity, several of them were detected in either genome. However, many had shown only partial similarity to those in the databases. For the MW-W600-10 a cluster with similarity to showdomycin (52% similarity) was detected. Few clusters with a lower similarity to potential antifungal compounds were present as well, including herboxidiene (6% similarity) and WS9326 (7% similarity). For S-2, two clusters similar to surugamide A/D (57% and 61% similarity), and singular clusters for fredericamycin A (100% similarity), antimycin (100%), and levorin A3/candicidin/nystatin (100% similarity) were detected. Among those showing a low level of similarity, valinomycin/montanastatin (13%) and herboxidiene (12%) were detected. Both strains have SGR PTMs encoding a cluster with 100% and 83% similarities for MW-W600-10 and S-2, respectively. Showdomycin is a uridine analog and has been shown to have antibacterial [65] along with antitumor activity [66,67]. Herboxidiene, found in both strains, is a wide-range metabolite that targets mRNA splicing [68]. WS9326 is NRPS with a rare N-terminal cinnamoyl moiety and has been shown to exhibit anti-*Candida* activity by the inhibition of isocitrate lyase [69]. Surugamide A was shown before to have antifungal activity and was produced by *S. albiflavus* J1074 to which S-2 is closely related [70]. Fredericamycin A exhibits antitumor activity and is an inhibitor of topoisomerases [71]; it cannot be excluded that it can pose similar activities against fungal enzymes. Antimycins are mitochondrial respiratory inhibitors produced by *Streptomyces* and were shown to pose antifungal activity [72,73]. Candicidin and nystatin have been known for their antifungal activities for years [24,74,75]. SGR PTMs are a class of polycyclic tetramate macrolactams initially found in *S. griseus* and exhibit a number of activities, including antifungal [76]. They are compounds widely distributed among *Streptomyces* genus [77]. Valinomycin is a peptide antibiotic, which was shown to be effective against *Candida, Cryptococcus albidus*, and plant pathogen *Botrytis cinerea* [78,79,80]. Furthermore, both strains pose BGCs, encoding several desferrioxamine B siderophores (100% similarity). Moreover, MW-W600-10 has clusters encoding coelichelin (90% similarity) and collismycin A (95% similarity), an iron-scavenging derivative of SF2768 [81], while S-2 has clusters encoding chalkophore SF2768 (66% similarity), which was shown to mediate copper acquisition in *S. thioluteus* [82]. These compounds can be used by strains to scavenge microelements—iron and copper, and, as such, have indirect antifungal activity [83].

Although *Streptomyces* genomes have a high number of biosynthetic gene clusters, many of them are dormant or not expressed under laboratory conditions [37,38,39]. A number of solutions could be potentially employed to induce the expression of dormant clusters. They involve genetic manipulation of the regulatory network of the clusters [39], co-culture approaches with other microorganisms [38,84], and stimulations by culture supplementation [85,86]. Work by Colombo et al. [87] has shown the influence of the culture medium on the antifungal activity of 20 *Streptomyces* strains against six strains of *Fusarium*. It would be interesting to investigate whether culture conditions have an influence on the antimicrobial activity of *Streptomyces* from coal-related environments. We have attempted to obtain insight about clusters and compounds responsible for the antifungal activity of strains by examining changes in the expression of selected BGCs—seven for MW-W600-10 (one cluster targeted by two primer sets) and five for S-2 (one cluster targeted by two primer sets). The results varied a great deal between clusters and between repeated experiments, rendering the majority of results not statistically significant. The reason for such a situation could be the complex regulation of secondary metabolism that is present in *Streptomyces*. Regardless, the results provide clues on the genes that might contribute to antifungal activity. For the S-2 strain, there is an increase in expression of two of the analyzed clusters observed at the 3rd day and onward. Moreover, the fungal presence further stimulated the expression. These clusters show similarity to surugamide A/D (57%) and fredericamycin A (100%). The upregulation of the second cluster was the highest of those analyzed, interestingly. As mentioned above, fredericamycin A poses antitumor activity, and it would be interesting to see whether the secondary metabolites produced by this strain show activity against cancer cells. For the MW-W600-10, there was no such high increase in expression of any analyzed genes. However, it cannot be excluded that other clusters that have not been studied here play a role. Nevertheless, an 8.8-fold increased expression of one of the clusters was observed at the 7th day of culture. Peculiarly, this cluster was predicted to be NRPS but shows no similarity to other clusters in the database. It might encode a new bioactive compound. The obtained results point to the importance of studying strains from extreme and previously neglected environments.

## 4. Materials and Methods

### 4.1. Isolation and Culture of the Strains

Underground water from the Upper Silesian Coal Basin was collected, taking advantage of the ongoing exploitation of coal seams in the region. The sampling was performed at the active hard-coal coal mine in the Upper Silesian Industrial Region in southern Poland. The coal-bearing Late Carboniferous rocks at the sampling site belong to the Mudstone series of sediments [88,89]. A sample of collective coal mine water from the shaft (depth of 665–850 below ground level (bgl)) was collected into a sterile bottle at a depth of 665 m by the authors. A total of 500 mL of water was collected from water intake at the underground pump station. The pump station regulates water removal from the coal mine shaft. After collection, the sample was stored at room temperature and delivered to the laboratory at the University of Silesia in Katowice, where it was immediately used for inoculation. It took approx. 4 h between sample collection and the beginning of sample processing in the laboratory. For inoculation, 200 μL of the sample was spread on a minimal salt medium supplemented with hard coal [43,90] (MSMA-C2), Reasoner’s 2A agar (R2A), nutrient agar (NA), and potato-dextrose agar (PDA) plates. Plates were incubated at 26 °C in the dark and investigated daily for the microbial growth. The appearing colonies were streak-inoculated on the fresh media plates and kept under the same conditions in order to obtain pure cultures. The MW-W600-10 strain was isolated on the PDA medium. The *Streptomyces* sp. S-2 and *N. oryzae* roseF7 were from the collection of the University of Silesia in Katowice, where their isolation and culture conditions have been described previously [43]. The *F. culmorum* DSM62188 was obtained from the German Collection of Microorganisms and Cell Cultures and provided to us by Dr. Małgorzata Rudnicka from the University of Silesia. The strain was cultured on the PDA medium at 26 °C in the dark with refreshing passage on the fresh medium every 10 days.

### 4.2. Whole-Genome Sequencing of the MW-W600-10 Strain and Phylogeny

The *Streptomyces* sp. MW-W600-10 was spread-inoculated on the PDA medium and incubated for 10 days, as described above. Then, the cells were scraped from the plate and sent for sequencing to MicrobesNG (Birmingham, UK). The whole genome sequencing was performed using an Illumina MiSeq platform (San Diego, CA, USA) with 2 × 250 bp paired-end reads. The sequencing results were processed via the standard MicrobesNG analysis pipeline (Birmingham, UK).

For nanopore sequencing, the genomic DNA was purified from a 100 mL PDA culture. The bacterial mass was harvested and lyophilized, and high-quality genomic DNA was extracted, as previously described [91]. Then, 400 ng of HMW genomic DNA was barcoded with the SQK-LSK109 kit (Nanopore Technologies, Oxford, UK) according to the manufacturer’s instructions using the EXP-NBD104 barcodes and was loaded onto the R94 flow cell (Nanopore Technologies, Oxford, UK). The data were acquired by MinKNOW (Nanopore Technologies, Oxford, UK), and base calling and demultiplexing were performed with Guppy v 4.2.2 (Nanopore Technologies, Oxford, UK). The reads were subjected to trimming with Filtlong v 0.2.0 (https://github.com/rrwick/Filtlong; accessed on 9 March 2020) and then assembled using Miniasm v 0.3 and minimap2 v 2.17 [92]. Finally, the assembly was polished by Racon v.1.3.3 [93] and Medaka v1.0.1 (https://github.com/nanoporetech/medaka; accessed on 9 March 2020). The qualities of the assembled genomes were evaluated, and gene groups were predicted using the Comprehensive Genome Evaluation tool at the PATRIC server (accessed on 31 March 2021) [94].

The phylogenetic tree of the MW-W600-10 strain was created by aligning core proteomes from the strain and 49 genomes using M1CR0B1AL1Z3R [95]. A full list of strains used for phylogenetic analysis with basic statistics is encompassed in Table S2. The poorly aligned blocks were removed using the Gblocks tool (version 0.91b) [96], resulting in an alignment of 16403 amino acids for each strain. A maximum-likelihood phylogenetic tree was calculated using IQ-TREE software [97] and validated by 1000 bootstraps.

### 4.3. Putative Biosynthetic Gene Clusters Analysis

The detection of biosynthetic gene clusters in *Streptomyces* genomes was performed using the antiSMASH tool, version 5.2.0 [98,99,100,101] in relaxed detection mode. The genomes used for the detection were close relatives of the S-2 and MW-W600-10 strains, as well as strains with a high number of BGCs, as reported in the literature. Selected groups of secondary metabolites were counted, and clusters classified as different than the selected group were pooled together as Other. As a single region detected by antiSMASH might comprise more than one cluster, results for each genome were investigated manually. The “neighboring clusters” were not counted; instead, each cluster was counted as separate. As the same large cluster might be responsible for the synthesis of a variety of compounds depending on the regulatory genes [102], interleaved clusters were counted as separate clusters. A cluster was considered a hybrid when antiSMASH classified it as a hybrid cluster. A tree of 35 *Actinobacteria* genomes was generated by retrieving 31 single-copy, housekeeping genes from each genome using AmphoraNet [103]. The genes that were not found in all of the analyzed genomes, as well as those that were fragmented, were excluded from analysis. This resulted in a set of 12 single-copy, housekeeping genes for each genome (*frr*, *infC*, *pgk*, *pyrG*, *rplA*, *rplC-F*, *rplK*, *rplL*, *rplP*, *rplS*, *rpoB*, *rpsE*, *rpsI*, *rpsM*, and *rpsS*). A list of genomes with accession numbers used for antiSMASH analysis can be found in supplementary Table S3. The alignment of each gene was performed in Unipro Ugene software [104] using the MUSCLE algorithm. Then, alignments were concatenated and poorly aligned blocks were removed using Gblocks (version 0.91b) [96], resulting in 2143 amino acids for each genome. A maximum-likelihood tree was calculated using the PhyML tool in Unipro Ugene software [104]. The quality of the tree was asserted by 1000 bootstraps. The tree was visualized using the iTOL web service [105].

### 4.4. Antifungal Assay

An antifungal assay was performed on the Mueller–Hinton agar (MHA) medium. For that, *Streptomyces* strains cultured for 10 days were inoculated as two 30 mm streaks at distances of 20, 25, or 30 mm from the center of the plate. Then, a puck of agar 8 mm in diameter with fungal mycelium cut-out from 10 days of fungal culture on the PDA plate was placed at the center of the plate. The fungal inoculation on the plates was simultaneous or after 3, 7, or 14 days of pre-culture of *Streptomyces* strains. Pictures of the plates were taken at 3, 7, and 14 days of co-culture. As a control, MHA plates inoculated simultaneously or after pre-incubation with fungal mycelium only were used. The fungal growth distance toward the *Streptomyces* streak (STR) or plate wall (WALL) was measured using Fiji software [106].

### 4.5. RNA Isolation and RT-qPCR

The expression of selected biosynthetic gene clusters from *Streptomyces* sp. MW-W600-10 and *Streptomyces* sp. S-2 strains was examined by RT-qPCR. That strain was spread-inoculated on 1/3 of the MHA plate surface and incubated as a single culture or in a co-culture with *F. culmorum* DSM62188 at 26 °C for 3, 6, 7, or 14 days. For the co-culture, a 8 mm puck of agar with fungal mycelium was placed 25 mm from the edge of the inoculation zone of the strains. The co-cultures were simultaneous or with the *Streptomyces* pre-culture for 3 or 7 days. After the incubation period, *Streptomyces* cells were scraped and RNA was isolated using the Beat-bead Total RNA Mini kit (A&A Biotechnology, Gdańsk, Poland) according to the manufacturer’s instructions. Two plates were pooled for each sample and, for each setup, purification from at least 3 biological independent experiments was performed. Purified RNA was treated with 2 U of DNase I (A&A Biotechnology, Gdańsk, Poland). cDNA amplification was performed on 600 ng of RNA using the ReverseAid First Strand cDNA Synthesis Kit (ThermoFisher Scientific, Waltham, MA, USA) according to the manufacturer’s protocol. A random hexamer primer was used for the first strand synthesis. The resulting cDNA was used for real-time quantitative PCR (RT-qPCR) performed using FastStart Essential DNA Green Master (Roche, Basel, Switzerland). Each reaction comprised 5 μL of SYBR Green Master Mix, 0.5 μL of each primer (10 μM), 2 μL of cDNA, and 2 μL of water. RT-qPCR was performed using a LightCycler 96 device (Roche, Basel, Switzerland) with the following program: preincubation at 98 °C for 600 s, followed by 40 cycles of 98 °C for 10 s, 59 °C for 20 s, and 72 °C for 30 s, and a single signal reading at 81 °C for 1 s. Cycles were followed by melting curve determination: 98 °C for 10 s, 65 °C for 60 s, followed by continuous signal reading until 98 °C with a 0.5 °C/s temperature ramp. The expression of RNA polymerase principal sigma factor gene, *hrdB*, was used as a reference. A list of clusters and primers used in the analysis can be found in Table S4. The relative expression of clusters was calculated using the delta-delta Cq method (R = 2^−^^ΔΔCq^; where R is the expression ratio and Cq is a reaction cycle in which the read signal exceeds the background) according to Livak and Smittgen [107]. As a control, the average ΔCq of 3 days of *Streptomyces* culture was used for each setup.

### 4.6. Statistical Analysis

The results of the growth distance measurement were analyzed for statistical significance using the one-way ANOVA test followed by Tukey’s post-hoc test. For each co-culture, 5 plates were prepared and, for every control, 3 plates were used. For statistical analysis of the clusters’ relative expression, outliers were detected and removed from each experimental setup dataset using Grubb’s test (*Q* = 5%). Significance of differences was tested using one-way ANOVA followed by Tukey’s post-hoc test. All statistical analyses were performed using GraphPad Prism version 9.1 software (GraphPad, San Diego, CA, USA).

## 5. Conclusions

We isolated the new *Streptomyces* sp. MW-W600-10 strain from underground coal mine water sampled at 665 m bgl. The strain is related to *S. fimicarius* and *S. baarnensis*. It shows strong antifungal activity against plants’ pathogenic fungi strains. We tested the influence of strain pre-incubation on the antifungal activity and showed that even if the effect is not strong, when the examined strain is co-cultured simultaneously, it still may show activity as the bioactive metabolites have to accumulate to reach the activity threshold, which is an important thing to remember in the search of natural biocontrol agents. Genome mining revealed the strain biosynthetic potential as its genome encompasses 39 putative BGCs, 13 of which does not show similarity to any compounds in the databases, potentially encoding new compounds. Moreover, we revised the biosynthetic potential of previously isolated *Streptomyces* sp. S-2, which also has strong antifungal properties. The revised analysis showed a putative BGCs number lower than previously detected, indicating the influence of the employed sequencing method on detection. This highlights the importance of the sequencing approach used for the evaluation of strains of biosynthetic potential. The combination of Nanopore long-reads and high-precision Illumina sequencing allowed for the better prediction of BGCs and the reduction in the detection of long clusters as separate ones. Among the detected clusters in either strain, the number showed similarity to clusters encoding antifungal metabolites. Those showing a similarity above 50% to known clusters included SGR PTMs, showdomycin, levorin A3/candicidin/nystatin, surugamid A/D, fredericamycin A, and antimycin. Additionally, BGCs for several siderophores were detected, desferrioxamine B, SF2768, and coelichelin, which can have an indirect antifungal effect through scavenging microelements. Finally, we examined the response of both strains to fungus by studying the change in expression of selected BGCs in co-cultures with fungus. It showed an increased expression of herboxidiene/atratumycin, SGR PTMs, and unknown NRPS for the MW-W600-10 strain, while fredericamycin A and surugamide A/D were upregulated in the S-2 strain, pointing to the compounds involved in the strains’ antifungal response. On the other hand, RP-1776, collismycin A, and herboxidiene for MW-W600-10, and herboxidiene and levorin A3/candicidin/nystatin for S-2 were downregulated. It is important to note that some of the analyzed clusters could have a high native expression, and they could contribute to the antifungal activity even though they were not upregulated/downregulated in the relative expression analyses. It is an issue that should be addressed in future experiments by both the examination of their native expression of the clusters, as well as the identification of metabolites produced by both strains. The identification can also lead to the discovery of novel compounds. In future experiments, it will be important to test the activity of separate compounds. This will allow the specification of compounds with antifungal activity. Separated compounds could also be tested for other properties, e.g., cytotoxicity, or antitumor activity. The examination of the strains’ interaction with plants will be of high importance in the scope of using the strains as natural biocontrol agents. Finally, future experiments should also focus on understanding the regulatory network of BGCs to increase the chance for cluster expression and, as such, the full exploitation of the strains’ potential. Overall, these results show that coal-related environments, including underground coal mine environments, can be a source of new strains and compounds with biotechnological potential. Future studies of strains from such niches would allow us to fully elucidate their biotechnological potential.

## Figures and Tables

**Table 1:** Next-generation sequencing of statistical data and comparison of genome features of the coal-related environment of *Streptomyces* strain’s draft genomes.

**General Genome Statistics**		
	***Streptomyces* sp. S-2**	***Streptomyces* sp. MW-W600-10**
Number of contigs	3	4
N50	4,185,286 bp	5,533,251 bp
Genome Length	7,243,498 bp	8,432,369 bp
G + C content	73.28%	71.70%
Completeness	100%	99.8%
CDS	6635	7738
tRNA	67	66
rRNA	21	18
**Protein Features**		
Hypothetical proteins	2269	2603
Proteins with functional assignments	4366	5135
Proteins with EC number assignments	1185	1246
Proteins with GO assignments	1032	1083
Proteins with pathway assignments	925	970
**Speciality Genes**		
Antibiotic resistance [CARD]	2	5
Antibiotic resistance [NDARO]	2	5
Antibiotic resistance [PATRIC]	40	60
Drug target [DrugBank]	7	7
Drug target [TTD]	-	1
Transporters [TCBD]	30	40
Virulence factors genes [PATRIC_VF]	2	2
Virulence factor genes [Victors]	1	1

Databases used for gene analysis are indicated in square brackets; CDS—coding sequences; EC—enzyme commission; GO—gene ontology.

**Table 2:** Comparison of BGCs composition of coal-related *Streptomyces* strains and their closest phylogenetical counterparts.

BGCs Type	Number of Clusters			
	*Streptomyces* sp. S-2	*S. Albidoflavus* J1074	*Streptomyces* sp. MW-W600-10	*S. Fimicarius* NRRL ISP-5322
NRPS	8 (2)	5 (2)	7 (1)	14 (5)
NRPS-like	1	1	0	1
T1PKS	0	1 (1)	1 (1)	2 (1)
T2PKS	1	0	0	2
T3PKS	1	1	2	2
PKS-like	0	0	1 (1)	1
Bacteriocin	3 (3)	4 (3)	2 (1)	2 (2)
Siderophore	2	2	2	2
Lanthipeptide	2 (1)	2	6 (4)	4 (2)
Terpene	4 (1)	4	4 (1)	6 (2)
Ectoine	1	1	3	2
HgIE-KS	0	0	1	2 (2)
Butyrolactone	0	0	2 (1)	2
Melanin	0	0	1	0
Lassopeptide	0	0	1	0
Hybrid	5 (1)	5	4 (1)	0
Other	0	1	2 (2)	7

NRPS—Nonribosomal peptide synthetases; T1PKS—type 1 polyketide synthetases; T2PKS—type 2 polyketide synthetases; T3PKS—type 3 polyketide synthetases; PKS—polyketide synthetases; hgIE-KS—heterocyst glycolipid synthase-like PKS. Values in brackets indicate the number of clusters nonsimilar to those in the database.

## Data Availability

All data generated during this study are available in the paper or as supplementary materials. Genome sequences are stored in NCBI under accession numbers: *Streptomyces* sp. S-2—WMKI00000000 (version WMKI02000000) and *Streptomyces* sp. MW-W600-10—JAGTPS000000000 (version JAGTPS010000000).

## Supplementary Materials

The following are available online at https://www.mdpi.com/article/10.3390/ijms22147441/s1.
