# Supplementary material for: Antifungal Activity and Biosynthetic Potential of New Streptomyces sp. MW-W600-10 Strain Isolated from Coal Mine Water
Source: Int J Mol Sci. 2021 Jul 12;22(14):7441. doi: 10.3390/ijms22147441 (PMC8303363; doi:10.3390/ijms22147441)
Supplement: Supplementary file 1 [file ijms-22-07441-s001.zip › ijms-1253909-supplementary.pdf]

Supplementary Materials

Antifungal activity and biosynthetic potential of new *Streptomyces* sp. MW-W600-10 strain isolated from coal mine water.

Piotr Siupka [1,*], Frederik Teilfeldt Hansen [2], Aleksandra Schier [1], Simone Rocco[1], Trine Sørensen[2], Zofia Piotrowska-Seget[1]

[1] Faculty of Natural Sciences, Institute of Biology, Biotechnology and Environmental Protection, University of Silesia in Katowice, 40032 Katowice, Poland; aleksandra.kaszyca@us.edu.pl (A.S.); simone.rocco@edu.unito.it (S.R.); zofia.piotrowska-seget@us.edu.pl (Z.P.-S.)

2 Faculty of Engineering and Science, Department of Chemistry and Biosciences, University of Aalborg, 9220 Aalborg, Denmark; jft@bio.aau.dk (F.T.H.); trso@bio.aau.dk (T.S.)

* Correspondence: piotr.siupka@us.edu.pl

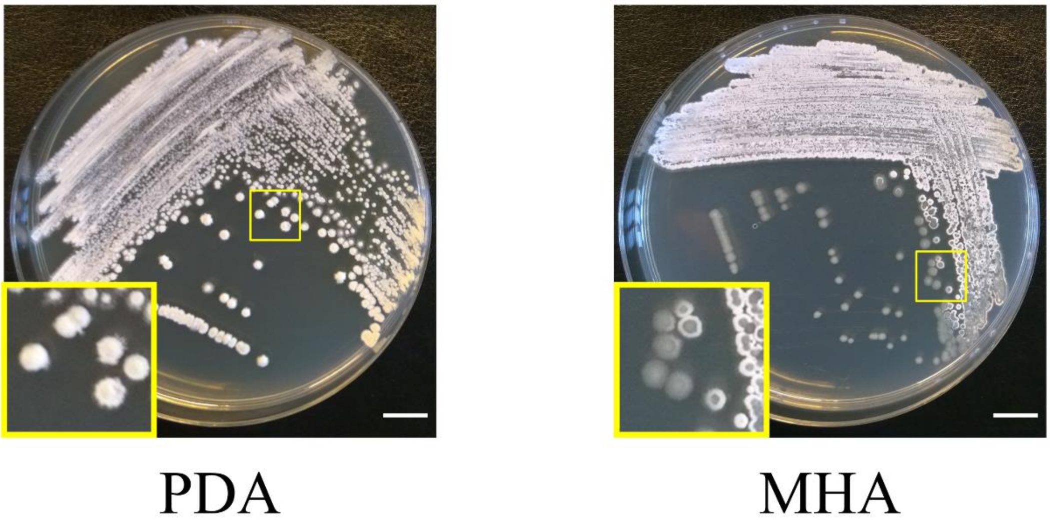

Figure S1. Morphology of *Streptomyces* sp. MW-W600-10 colonies during growth on PDA and MHA plates. Pictures represent 10 days culture, area showed in magnification marked by yellow box, scale bar represents 10 mm.

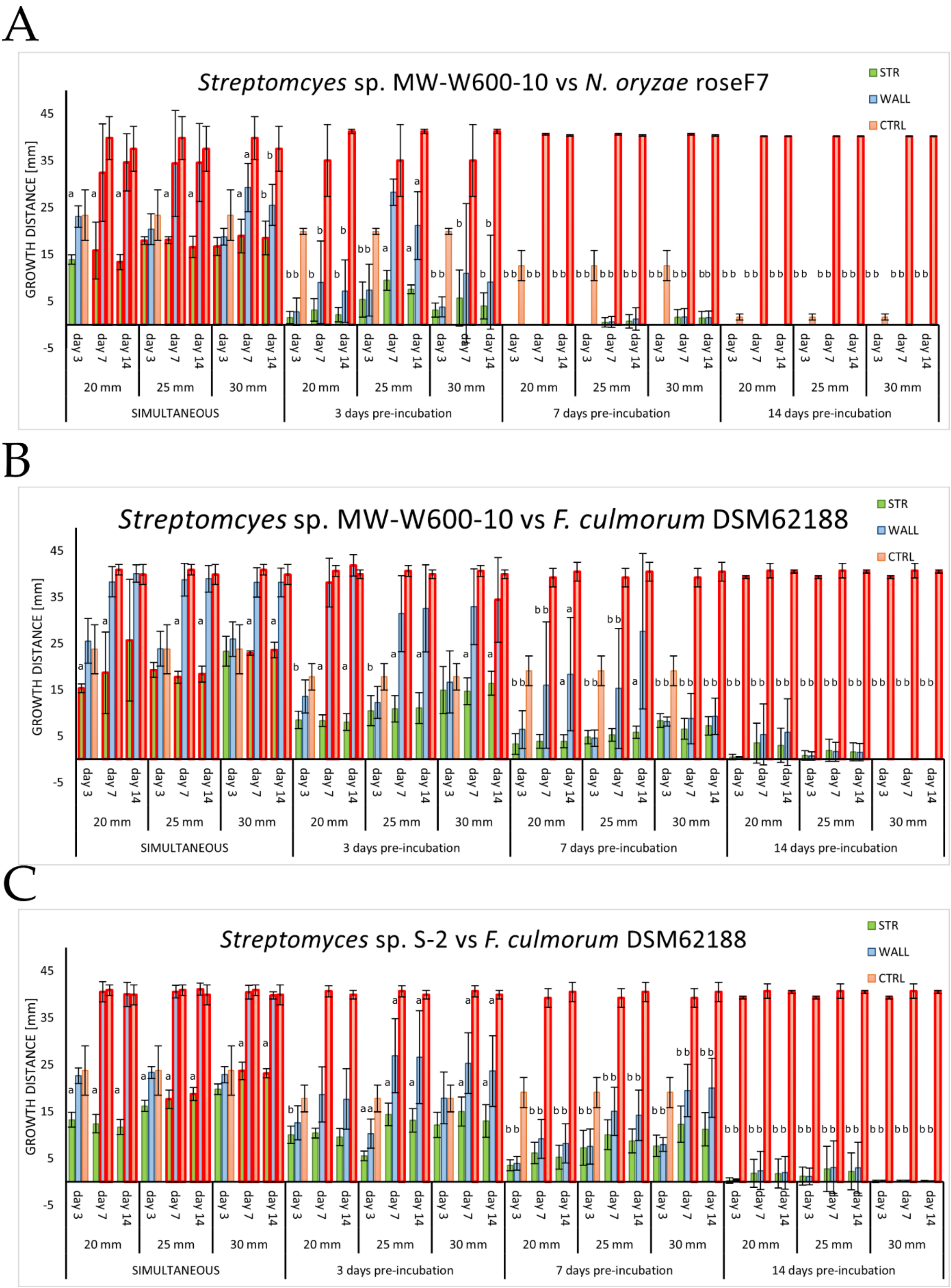

Figure S2. Inhibition of fungal growth by coal related Streptomyces strains. **A)** Effect of co-culture with MW-W600-10 strain on the growth of *Nigrospora oryzae* roseF7; **B)** Effect of co-culture with MW-W600-10 strain on the growth of *Fusarium culmorum* DSM62188; **C)** Effect of co-culture with S-2 strain on the growth of *F. culmorum* DSM62188. Charts comparing the growth of fungal mycelium toward *Streptomyces* streak colonies (STR), plate wall (WALL) and fungal growth on control plates (CTRL) for every experimental conditions. For each experimental set-up 5 biological repeats were performed for co-

cultures and 3 repeats for controls. Red border of the bars indicate fungus reaching plate wall or bacterial streaks by the day of measurement. Error bars represent standard deviation of mycelial growth in independent biological experiments. Statistically significant differences between measurements analyzed for each experimental setup separately are marked by ‘a’ – difference between all measurements or by ‘b’ – difference between measurement and CTRL only (one-way ANOVA, followed by Tuckey’s post hoc test; $p \leq 0.05$)

Table S1. Putative biosynthetic gene clusters in *Streptomyces* sp. S-2 and *Streptomyces* sp. MW-W600-10.

***Streptomyces* sp. S-2**						
**Cluster**	**Region**	**Type**	**span**		**most similar**	**similarity**
			**from**	**to**		
cluster 1	1.1	NRPS	2	47,790	surugamide A/ surugamide D	57%
cluster 2	1.2	NRPS	301,932	349,682	-	-
cluster 3	1.2	T2PKS	342,447	414,908	fredericamycin A	100%
cluster 4	1.3	NRPS	1,113,660	1,169,885	mannopeptimycin	51%
cluster 5	1.4	lantipeptide	1,196,700	1,219,279	SAL-2242	100%
cluster 6	1.5	hybrid - LAP/thiopeptide	1,545,106	1,577,578	fluostatins M-Q	4%
cluster 7	1.6	terpene	2,072,093	2,092,132	julichrome Q3-3 / julichrome Q3-5	25%
cluster 8	1.7	siderophore	2,676,162	2,691,264	ficellomycin	5%
cluster 9	1.8	NRPS	2,784,877	2,844,960	dechlorocuracomycin	16%
cluster 10	1.9	bacteriocin	3,090,781	3,100,551	-	-
cluster 11	1.1	bacteriocin	3,507,352	3,517,567	-	-
cluster 12	1.11	terpene	3,582,638	3,607,798	hopene	76%
cluster 13	1.12	hybrid - T1PKS/NRPS	3,643,598	3,693,008	SGR PTMs	83%
cluster 14	1.13	NRPS	3,849,935	3,905,582	valinomycin / montanastatin	13%
cluster 15	1.13	terpene	3,903,732	3,923,884	-	-
cluster 16	1.14	NRPS	3,942,908	3,989,201	diisonitrile antibiotic SF2768	66%
cluster 17	2.1	hybrid - T1PKS/NRPS-like	21,824	85,197	-	-
cluster 18	2.1	hybrid - T1PKS/NRPS	76,674	141,322	antimycin	100%
cluster 19	2.1	lanthipeptide	79,074	100,556	-	-
cluster 20	2.1	hybrid - T1PKS/NRPS-like	139,741	289,799	levorin A3 / C06690 / FR-008-III / candicidin A	100%
cluster 21	2.2	T3PKS	298,571	339,668	herboxidiene	12%
cluster 22	2.3	terpene	433,674	461,697	Isorenieratene	75%
cluster 23	2.3	bacteriocin	453,444	463,556	-	-
cluster 24	2.4	NRPS-like	483,827	527,053	indigoidine	80%
cluster 25	2.5	ectoine	1,215,008	1,225,406	ectoine	100%
cluster 26	2.6	siderophore	2,120,931	2,132,751	desferrioxamin B	100%
cluster 27	2.7	NRPS	2,359,555	2,402,406	-	-
cluster 28	2.8	NRPS	2,867,182	2,916,962	surugamid A / surugamid D	61%
***Streptomyces* sp. MW-W600-10**						
**Cluster**	**Region**	**Type**	**span**		**most similar**	**similarity**
			**from**	**to**		
cluster 1	1.1	butyrlactone	63,409	72,524	coelimycin P1	16%
cluster 2	1.2	NRPS	193,748	275,322	atratumycin	13%
cluster 3	1.2	T3PKS	270,607	311,451	alkylresorcinol	100%
cluster 4	1.3	melanin	372,163	382,660	istamycin	4%
cluster 5	1.4	NRPS	506,674	549,608	-	-
cluster 6	1.5	bacteriocin	623,922	633,435	tetronasin	3%
cluster 7	1.6	hybrid (NRPS-T1PKS)	649,631	697,969	SGR PTMs	100%
cluster 8	1.7	terpene	812,186	838,352	hopene	69%
cluster 9	1.8	ectoine	1,226,548	1,235,998	kosinostatin	11%
cluster 10	1.9	bacteriocin	1,504,970	1,516,319	-	-

cluster 11	1.10	hybrid (NRPS-T1PKS)	1,538,919	1,593,964	collismycin A	85%
cluster 12	1.11	siderophore	1,805,282	1,819,960	ficellomycin	3%
cluster 13	1.12	terpene	2,239,900	2,260,907	-	-
cluster 14	1.13	lanthipeptide	2,612,251	2,632,686	AmfS	100%
cluster 15	1.14	T1PKS	2,718,995	2,762,066	-	-
cluster 16	1.14	NRPS	2,722,521	2,771,911	WS9326	7%
cluster 17	1.15	lanthipeptide	3,400,142	3,421,928	-	-
cluster 18	1.16	lassopeptide	3,543,089	3,565,858	keywimysin	100%
cluster 19	1.17	ectoine	3,684,826	3,695,168	showdomycin	52%
cluster 20	1.17	butyrlactone	3,689,418	3,700,168	-	-
cluster 21	1.18	NRPS	3,701,430	3,788,526	RP-1776	51%
cluster 22	1.18	arylpolyene	3,758,449	3,798,852	-	-
cluster 23	1.18	ladderane	3,759,585	3,800,837	-	-
cluster 24	1.19	lanthipeptide	3,902,089	3,924,912	divergolide A / divergolide B / divergolide C / divergolide D	6%
cluster 25	1.20	PKS-like	4,235,964	4,276,932	-	-
cluster 26	1.21	lanthipeptide	4,415,530	4,439,359	-	-
cluster 27	1.22	lanthipeptide	4,886,102	4,911,525	-	-
cluster 28	1.22	NRPS	4,905,952	4,970,600	scabichelin	20%
cluster 29	1.23	hybrid (LAP-thiopeptide)	5,184,236	5,216,738	-	-
cluster 30	2.1	terpene	332,887	352,724	steffimycin D	19%
cluster 31	2.2	hglE-KS	672,064	720,670	borrelidin	9%
cluster 32	2.3	T3PKS	982,221	1,023,339	herboxidiene	6%
cluster 33	2.4	NRPS	1,078,578	1,126,456	coelichelin	90%
cluster 34	2.4	NRPS	1,124,789	1,179,680	streptobactin	94%
cluster 35	2.5	terpene	1,243,714	1,265,927	geosmin	100%
cluster 36	2.6	hybrid (NRPS-T1PKS)	1,278,162	1,330,650	balhimycin	8%
cluster 37	3.1	siderophore	59,182	70,960	desferrioxamin B	100%
cluster 38	3.2	lanthipeptide	143,641	167,009	-	-
cluster 39	3.3	ectoine	1,226,847	1,237,275	ectoine	100%

Table S2. List of strains used for phylogenetical analysis with basic statistics for genomes.

Strain	GenBank Accessions	Genome Length	GC Content	PATRIC CDS	RefSeq CDS	Isolation Source
Streptomyces albidoflavus J1074	CP004370	6841649	73.32	6120	5832	
Streptomyces alboflavus strain NRRL B-2373	JNXT00000000	9717375	72.2	8332		
Streptomyces albulus strain NK660	CP007574, CP007575	9372401	72.32	8793	8086	environment
Streptomyces albus DSM 41398	CP010519.1	8384669	72.64	6923	7330	soil
Streptomyces albus NRRL B-1335	ASM141854v1	6963283	72.54	6753		
Streptomyces ambofaciens ATCC 23877	CP012382, CP012383	8393598	72.19	7953	7801	soil
Streptomyces avermitilis strain NBRC 14893	BA000030.4	10484508	70.535835	10426	9908	
Streptomyces baarnensis strain NRRL B-2842	JNZV00000000	8162633	71.8	7294		
Streptomyces bingchenggensis BCW-1	CP002047	11936683	70.75	10313	10022	soil sample collected
Streptomyces cacaoi strain OABC16	VSKT01000000	8578932	73.35831	7350		marine sediment
Streptomyces cattleya DSM 46488	CP003219, CP003229	8095515	73.01	7543	7569	
Streptomyces cattleya NRRL 8057	FQ859184, FQ859185	8092553	73.01	7229	7483	
Streptomyces coelicolor A3(2)	AL645882, AL589148, AL645771	9054847	72	8325	8154	
Streptomyces collinus Tu 365	CP006259.1, CP006261.1, CP006260.1	8377286	72.5501	7336	7113	
Streptomyces cyaneogriseus subsp. *noncyanogenus* strain NMWT 1	CP010849	7762396	72.86	6922	5830	
Streptomyces davawensis JCM 4913	HE971709.1, HE971710.1	9555950	70.5935	8696	8616	
Streptomyces exfoliatus strain A1013Y	CP040244	7646296	71.588554	7234		soil
Streptomyces fimicarius strain NRRL ISP-5322	MUNB00000000	7819863	71.74	7739		soil
Streptomyces formicae strain KY5	CP022685	9611874	71.38	8393	8162	Kenya
Streptomyces fulvissimus DSM 40593	CP005080.1	7905758	71.5	7081	6925	
Streptomyces glaucescens GLA.O	CP009438.1, CP009439.1	7623774	72.91	6719	6567	soil
Streptomyces globisporus C-1027	AJUO00000000	7689864	71.6	6980		soil
Streptomyces griseus subsp. *griseus* NBRC 13350	AP009493	8545929	72.2	7294	7136	
Streptomyces griseus subsp. *griseus* strain ATCC 13273	CP032543	7238194	71.70149	6714		soil

Streptomyces halstedii strain NRRL ISP-5068	JOAZ00000000	7740838	71.9	6764		
Streptomyces hygroscopicus subsp. *hygroscopicus* NBRC 100766	BCAN00000000	10165415	71.88	9090		
Streptomyces hygroscopicus subsp. *jinggangensis* 5008	CP003275, CP003276, CP003277	10383684	71.84	9901	9108	
Streptomyces hygroscopicus subsp. *jinggangensis* TL01	CP003720.1, CP003722.1, CP003721.1	10077952	42.7065	9146	8878	
Streptomyces iranensis	LK022848, LK022849, LK022850, LK022851, LK022852, LK022853, LK022854	12130542	70.86	10394	9967	
Streptomyces koyangensis strain VK-A60T	CP031742	7220839	73.0269	6695	6318	soil
Streptomyces lividans 1326	APVM01000000	8496762	72.16328	8050	8081	soil
Streptomyces lividans TK24	CP009124	8345283	72.24	7749	7360	
Streptomyces lydicus A02	CP007699	9300149	70.7	8888	8518	
Streptomyces lydicus strain GS93	CP019457	8243179	72.01	7497		soil
Streptomyces mediolani strain NRRL WC-3934	JOJK00000000	8120903	71.4	7420		soil
Streptomyces nodosus strain ATCC 14899	CP009313	7714110	70.8	7170	5881	Orinoco river basin
Streptomyces pratensis ATCC 33331	CP002475, CP002476, CP002477	7656104	71	6866	6572	
Streptomyces rimosus strain ATCC 10970	CP023688	9361154	71.95971	8671	8107	soil
Streptomyces roseosporus NRRL 15998	ABYB00000000	7817295	71.3	6857	6902	
Streptomyces scabiei 87.22	FN554889	10148695	71.57	8977	8746	
Streptomyces sp. 769	CP003987.1, CP003988.1	10338286	71.6	9177	9553	soil
Streptomyces sp. MW-W600-10	JAGTPS000000000	8432369	71.70	7738		shaft's collective coal mine water
Streptomyces sp. PAMC26508	CP003990.1, CP003991.1	7630245	71.0645	6806	7073	
Streptomyces sp. S-2	WMKI00000000	7243498	73.28	6635		black soot
Streptomyces sp. SirexAA-E	ADFD01000000	7414440	71.75	6808	6357	
Streptomyces venezuelae ATCC 10712	FR845719.1	8226158	72.4	7409	7453	
Streptomyces vietnamensis GIM4.0001	CP010407.1, CP010408.1	9153777	71.99	8292	7356	
Streptomyces violaceusniger Tu 4113	AEDI00000000	10988130	70.91	9644	9485	
Micromonospora aurantiaca ATCC 27029	CP002162	7025559	72.9	6337	6222	soil
Micromonospora sp. ATCC 39149	GCA_000158815.1	6819904	72.3	5896	5633	soil

Table S3. List of strains used for biosynthetic gene clusters prediction by antiSMASH with basic statistics for each genome.

Genome Name	GenBank Accessions	Genome Length	GC Content	PATRIC CDS	RefSeq CDS	Isolation Source
Micromonospora aurantiaca ATCC 27029	CP002162	7025559	72.90	6337	6222	soil
Micromonospora echinospora DSM 43816	LT607413	7775586	72.31	6966	6449	soil
Streptomyces albus J1074	CP004370	6841649	73.32	6120	5832	
Streptomyces albus SM254	CP014485	7170504	73.34	6473	6180	copper-rich brine from underground mine
Streptomyces ambofaciens ATCC 23877	CP012382	8393598	72.19	7953	7801	soil
Streptomyces bingchenggensis BCW-1	CP002047	11936683	70.75	10313	10022	soil
Streptomyces coelicolor A3(2)	AL645882	9054847	72.00	8325	8154	soil
Streptomyces fildesensis So13.3	PYSU00000000	9475060	70.50		8586	Antarctic soil
Streptomyces fimicarius strain NRRL ISP-5322	MUNB00000000	7819863	71.74	7739		soil
Streptomyces formicae KY5	CP022685	9611874	71.38	8393	8162	from *Tetraponera penzigi* fungus growing ants
Streptomyces griseochromogenes ATCC 14511	CP016279	10813680	70.45	10278		soil
Streptomyces griseus S4-7	JYBE01000000	7628220	71.58	7220	6930	strawberry rhizosphere
Streptomyces griseus subsp. *griseus* NRRL WC-3066	LLZL01000000	7151148	73.41	6613	6118	forest soil
Streptomyces hygroscopicus subsp. *jinggangensis* 5008	CP003275	10383684	71.84	9901	9108	Jinggang Mountain area of China
Streptomyces kanasensis ZX01	LNSV00000000	7026257	73.88	6814	5961	woods soil
Streptomyces lydicus 103	CP017157	8201357	72.22	7502	7036	soil
Streptomyces lydicus WYEC 108	CP029042	9125666	70.80	8388	7882	soil
Streptomyces olivoreticuli subsp. *olivoreticuli* ATCC 31159	CP031455	8809793	71.11	7923		soil
Streptomyces rapamycinicus NRRL 5491	CP006567	12700734	70.60	10393	10002	soil
Streptomyces roseosporus NRRL 11379	ABYX00000000	7881159	71.40	7079	7056	
Streptomyces silaceus ACCC40021	CP015588	8625867	72.09	7813	7258	root

Streptomyces sp. CC71	LOSR00000000	7888579	72.35	7612	6564	sediment from the Churince hydrological system
Streptomyces sp. FR-008	CP009802	7258031	73.32	6897	7090	protoplast fusion of two *Streptomyces strai ns*
Streptomyces sp. GBA 94-10	ASHF01000000	7224475	72.96	6566	6148	sponge
Streptomyces sp. GKU 895	MWJO00000000	8296413	70.73	8708	7635	roots of *Saccharum officinarum*
Streptomyces sp. ISID311	VOQD01000000	8187711	71.04	7479	7003	
Streptomyces sp. JS01	JPWW00000000	7799375	71.60	7139	6693	mangrove sediment
Streptomyces sp. KE1	LAYX01000000	6830045	73.43	6294	5650	healthy human skin surface
Streptomyces sp. MBRL 601	JXOM01000000	4508621	71.85	5994	2647	rhizome of Acorus calamus
Streptomyces sp. MW-W600-10	JAGTPS000000000	8432369	71.70	7738		shaft's collective coal mine water
Streptomyces sp. PTY087I2	LZRD00000000	8164109	71.50	7388	7035	*Styela canopus*, a tunicate collected
Streptomyces sp. PVA 94-07	ASHE01000000	7106149	73.05	6359	6004	sponge
Streptomyces sp. S-2	WMKI00000000	7243498	73.28	6635		black soot
Streptomyces sp. SM8	AMPN00000000	7152085	73.30	6352	6517	from *Haliclona simulans*
Streptomyces wadayamensis A23	JHDU00000000	7056006	73.30	5998	5463	plant tissue

Table S4. List of primers used for RT-qPCR study of BGCs expression.

Strain	Cluster (region)	Similarity (genes similarity in %)	Primers (5’-3’)	
Streptomyces sp. S-2	Cluster 1 (region 1.1)	surugamid A/D (57 %)	Forward	ATCAGCCTCCAGGTGGTCTC
			Reverse	GGATGCGTCTCGAAGAACCAG
	Cluster 3 (region 1.2)	fredericamycin A (100 %)	Forward	CTGAACGACCTGGACATGG
			Reverse	CACGTCCAGGCCGCCGGC
	Cluster 20 (region 2.1)	levorin A3/candidacin A/nystatin (100 % / 100 % / 73 %) [NRPS domain]	Forward	GAGATCTCACCACCGGTCAC
			Reverse	GCCGACCTACGCCTTCCAG
	Cluster 20 (region 2.1)	levorin A3/candidacin A/nystatin (100 % / 100 % / 73 %) [T1PKS domain]	Forward	GCTGTTCGAGGATGACGTG
			Reverse	CGGTGTGGCGGGTGTGATC
	Cluster 21 (region 2.2)	herboxidiene (12 %)	Forward	GTAGTCCTGGCCGTTGGTG
			Reverse	CCTCCAGGGCGCCGGACTC
	Cluster 27 (region 2.7)	unknown NRPS (-)	Forward	GGACAACGATGTCGCCTATG
			Reverse	CCACAGGGCGAGGAAGAGG
Streptomyces sp. MW-W600-10	Cluster 2 (region 1.2)	herboxidiene/atratumycin (11 % / 13 %)	Forward	GACCTGGTCGTCACCGAAC
			Reverse	CCTTGGGTTCGCCGGTGGAG
	Cluster 2 (region 1.2)	herboxidiene/atratumycin (11 % / 13 %)	Forward	CTGTTCGTCAACACCGTTCC
			Reverse	CGGATAGTTCTCCAGGGCCG
	Cluster 5 (region 1.4)	unknown NRPS (-)	Forward	GGGGTCGAGTGTGTACATGG
			Reverse	CATCCCTCACCCGCACCAAC
	Cluster 7 (region 1.6)	SGR PTMs (100 %)	Forward	GTTCCTCACCGTCGAAGTGC
			Reverse	CGGCTGAGCGGAAGCGGAC
	Cluster 11 (region 1.10)	collismysin A (85 %)	Forward	GATGATCGGCGTGTTCAAG
			Reverse	GGGGAGGTGGCGGTGGCAGG
	Cluster 21 (region 1.18)	RP-1776 (51 %)	Forward	TCTGGAAGAGCGGATGACG
			Reverse	GTGACGACGAGGCGCTGGAG
	Cluster 25 (region 1.20)	unknown PKS-like (-)	Forward	AGTACGCACTCGTGCTGGTC
			Reverse	GACGACCCGGTCGTTCCGGC
	Cluster 32 (region 2.3)	Herboxidiene (6 %)	Forward	GTTCTGCTCGCTGTGCTACC
			Reverse	GAAGTGGAACCCGGTGTCGC

REFERENCE GENE	-	hrdB	Forward	GATCACCCGCGCCATGGCC
			Reverse	GGACCTCGATGACCTTCTCGG
